# Modeling the persistence of 4CMenB vaccine protection against real world meningococcal B disease in adolescents

**DOI:** 10.1038/s41541-024-01025-5

**Published:** 2024-12-02

**Authors:** Lorenzo Argante, Ottavia Prunas, Duccio Medini, Ellen Ypma

**Affiliations:** 1 GSK, Siena, Italy; 2 Swiss Tropical and Public Health Institute, Basel, Switzerland; 3 GSK, Amsterdam, The Netherlands; 4 Present Address: Toscana Life Sciences Foundation, Siena, Italy

**Keywords:** Meningitis, Meningitis, Epidemiology, Epidemiology

## Abstract

The efficacy of the four-component 4CMenB vaccine is measured through the serum bactericidal antibody (SBA) assay on four meningococcal B (MenB) indicator strains. However, they are not epidemiologically relevant for disease, thus the real-world persistence of 4CMenB protection remains uncertain. Several mathematical models of waning immunity were fitted on longitudinal SBA data from persistence studies in adolescents, with up to eight years follow-up after 4CMenB priming vaccination. The best model was used to predict protection from indicator strains. MenB typing data from the United States were used to integrate antigen-level curves and predict the persistence of protection from real-world MenB strains, considering synergies between antigens. Models show that protection and its evolution varied by antigen and that 4CMenB likely elicits antibody-producing long-lived plasma cells. 4CMenB protection from real-world MenB disease persisted at 61.5% four years post-priming and 70.5% four years post-booster. This evidence could support decision-making on adolescent immunization programs.

## Introduction

Invasive meningococcal disease (IMD) caused by serogroup B *Neisseria meningitidis* bacteria (MenB) is a major cause of meningitis and sepsis, associated with rapid disease progression, high morbidity and mortality, and a risk of severe or long-term sequelae in survivors. While the disease primarily affects infants and young children, there is a second peak in incidence among adolescents, who often have the highest nasopharyngeal carriage rates, increasing the risk of transmission^1–3^.

A multicomponent vaccine (4CMenB, GSK) is indicated in several countries to prevent MenB IMD^2–6^. It contains three recombinant protein antigens, factor H binding protein (fHbp) variant 1.1, *Neisseria* adhesin A (NadA), and Neisserial Heparin Binding Antigen (NHBA), that are combined with outer membrane vesicles (OMVs) from New Zealand strain NZ98/254 expressing Porin A as immunodominant protein (PorA)^7^.

Vaccine protection is mediated by antibodies, that have a relatively short lifespan, as they are degraded within a few weeks or months and their concentration declines exponentially if not replenished. An essential immunological determinant for a longer persistence of protection following vaccination is the generation and proliferation of plasma cells, in particular of long-lived plasma cells, that can continue to release antibodies for several years or even decades^8,9^.

Vaccine-induced immune response against IMD is measured through its correlate of protection, the serum bactericidal assay with human complement (hSBA)^10,11^. Data on persistence of 4CMenB protection in adolescents are available from clinical trials with follow up data of 2–8 years^12–17^. However, these studies have different durations, blood samples have been collected at different timepoints, and participants were recruited in different geographies. Moreover, 4CMenB hSBA titers are measured on four specific MenB indicator strains, each strain exhibiting one, and only one, of the four antigens that constitute the vaccine and representing the protection elicited by such antigenic component, but the four indicator strains are not epidemiologically relevant. Real-world MenB strains can actually express none, one or more 4CMenB components at the same time^18^.

To overcome these limitations while establishing a link between antigen-indicator strains and real-world MenB strains, hSBA data were complemented with cross-sectional MenB typing data, processed through the Meningococcal Antigen Typing System (MATS) for a collection of 442 MenB strains that represent IMD epidemiology in the United States of America (USA)^19,20^. These MenB typing data are correlated to hSBA^18^ and have been shown to be stable over time^20^.

The objective of this analysis was first to find the mathematical model that best reproduces 4CMenB vaccine’s correlates of protection and their evolution over time in vaccinated subjects from different studies, to estimate the persistence of antigen-specific immunological response. Secondly, to integrate best model’s predictions with real-world MenB typing data, to obtain a comprehensive persistence curve of 4CMenB protection from real-world MenB disease.

## Results

### Bactericidal titers for different antigens are best fitted by different evolution models

**Fig. 1:**
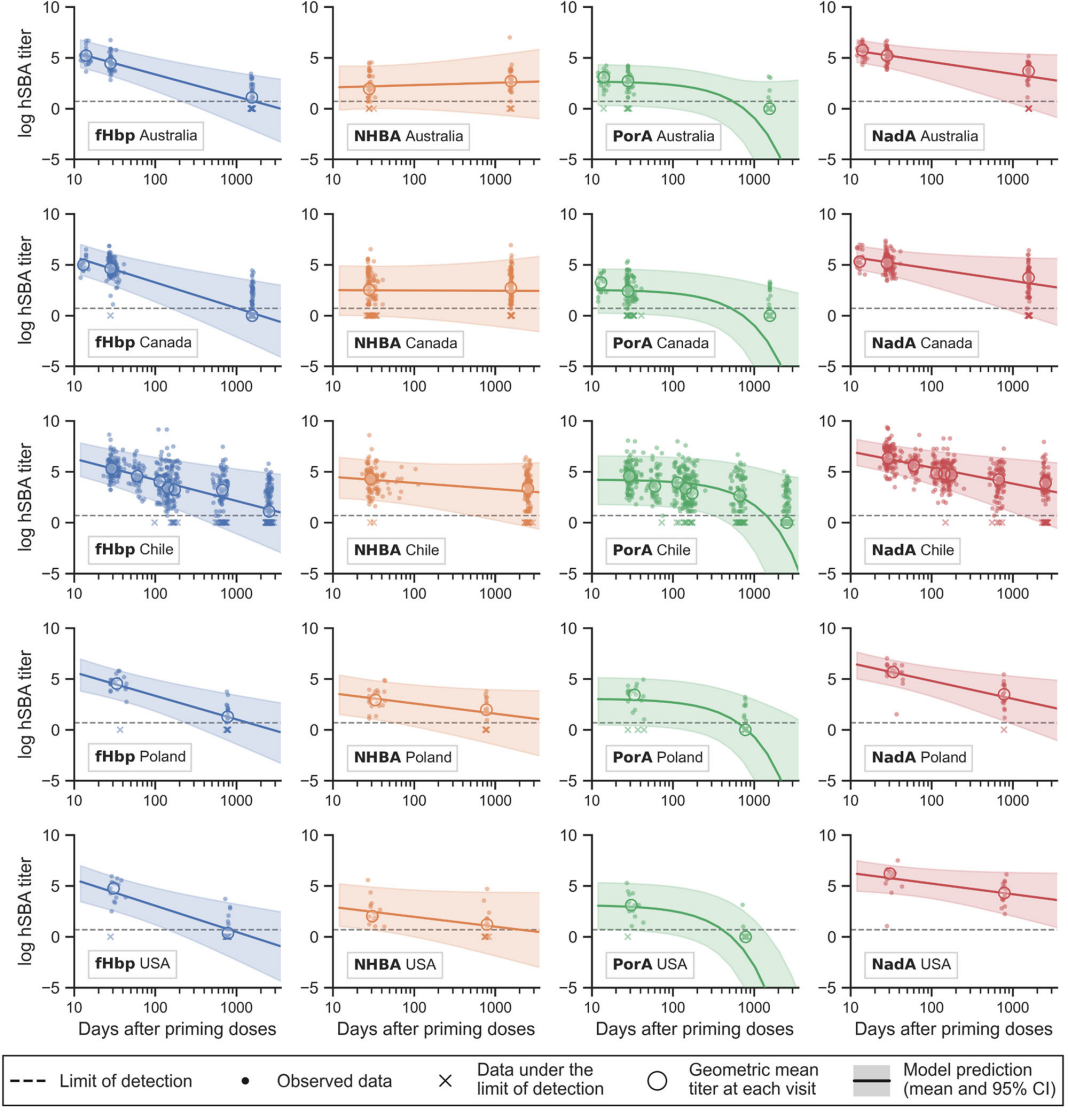
Bactericidal titers after priming vaccination and before any booster dose. Observed data and best model fits by country and antigen-indicator strain. Each panel reports observed data and best model’s Bayesian posterior predictive distributions for the logarithm of hSBA titer vs. time after second dose, for each antigen (by column) and for each country (by row). Single data points are presented as small dots, while their average at each visit is shown as bigger circles. Immunological readouts under the limit of detection (LOD, set to titer 2 and shown as a dashed horizontal line) are indicated with crosses and conventionally reported at titer 1 to facilitate visualization. The fitted curves are posterior means, with their 95% credible intervals shown as shaded areas. hSBA, human serum bactericidal antibody; fHbp, factor H binding protein; NHBA, Neisserial Heparin-Binding Antigen; PorA, Porin A; NadA, *Neisseria* adhesin A; USA, United States of America; CI, credible interval.

Several mathematical models of vaccine persistence were fitted to the data collected after priming and before booster vaccination, and their performances were compared (Table 1). The best performing model was a hierarchical model composed of distinct titer-time evolution models (power law models for fHbp, NHBA and NadA strains; exponential model for PorA strain) stratified by the countries where participants were recruited. Figure 1 shows the performance of the model in reproducing the post-priming data, that consist in bactericidal titers over time after second dose and before third dose by country and antigen-indicator strain. Together with the data, their respective best fitting curves and 95% credible intervals are reported, derived from the best performing model. In particular, best fits qualitatively show the differences in time-evolution for immune responses elicited by distinct antigens. Summaries of posterior distributions of the full set of best model parameters are reported in Supplementary Table S1.

**Table 1:** Comparison of 4CMenB vaccine persistence models

Variations with respect to best model	Type of variation	Difference in WAIC score (standard error)
Best model* (WAIC = -4340.6)	None	0 (0)
Slope variance stratified by antigen	*σ* stratification	-1.7 (5.8)
Intercept variance stratified by country	*σ* stratification	-14.0 (9.9)
Intercept variance not stratified by antigen	*σ* stratification	-22.8 (7.3)
Slope means not stratified by country	*μ* stratification	-22.8 (8.3)
NHBA evolution to exponential model	Evolution model	-62.2 (9.9)
PorA evolution to power law model	Evolution model	-88.1 (19.7)
Intercept mean not stratified by country	*μ* stratification	-90.8 (12.5)
fHbp evolution to exponential model	Evolution model	-165.3 (21.1)
NadA evolution to exponential model	Evolution model	-296.0 (24.2)
Non-hierarchical intercept	Model’s hierarchy	-577.3 (33.3)
Non-hierarchical slope	Model’s hierarchy	-582.3 (32.8)
Non-hierarchical intercept and slope	Model’s hierarchy	-1,377.4 (49.8)

The models are ordered by difference in WAIC score with respect to the best model. *Best model: combination of four evolution models for the four outcomes (power law for fHbp, NHBA and NadA titers; exponential for PorA titers), hierarchical in intercept parameters (a) and slope parameters (b and c), stratified by antigen and country. The reported differences in scores are calculated with respect to best model’s score (WAIC: -4,340.6).

NHBA, Neisserial Heparin-Binding Antigen; PorA, Porin A; fHbp, factor H binding protein; NadA, *Neisseria* adhesin A; WAIC, Widely Applicable Information Criterion; 4CMenB, four-component meningococcal serogroup B vaccine.

Table 1 reports the loss in the Widely Applicable Information Criterion (WAIC) score when changing one characteristic of the best fitting model while maintaining all the others. Performance always decreased when varying the antigens’ evolution models. The WAIC score decreased by 62.2 when using an exponential model instead of a power law model for NHBA; and by 88.1 when using a power law model instead of an exponential model for PorA. The performance was even worse with alternative evolution models for NadA or fHbp (Table 1, variations marked with “Evolution model”**)**.

The best model was hierarchical in both intercept and slope parameters. Non-hierarchical models had a drastically worse performance (WAIC differences well over 500, bottom of Table 1). Stratification by country of hierarchical means (“*μ* stratification” in Table 1) was also important to improve model fit (difference in WAIC scores from 22.8 to 90.8 when removing country strata from slopes and intercept means, respectively). Conversely, adding or removing antigen or country strata from hierarchical variances (*σ* parameters) had a negligible or small impact on model performance (“*σ* stratification” variations in Table 1 all achieved scores close to the best model).

### Long-term persistence is highest for NadA and NHBA, followed by fHbp and PorA

The best performing model was used to predict the persistence of protection provided by each antigen (i.e., for each antigen-indicator strain), evaluated as the proportion of participants having antigen-specific bactericidal titers ≥ 4 (protection threshold) at any time after the second priming dose. Predictions were done first by country, then merged into an overall estimate that inherently also takes into account geographical variability.

**Fig. 2:**
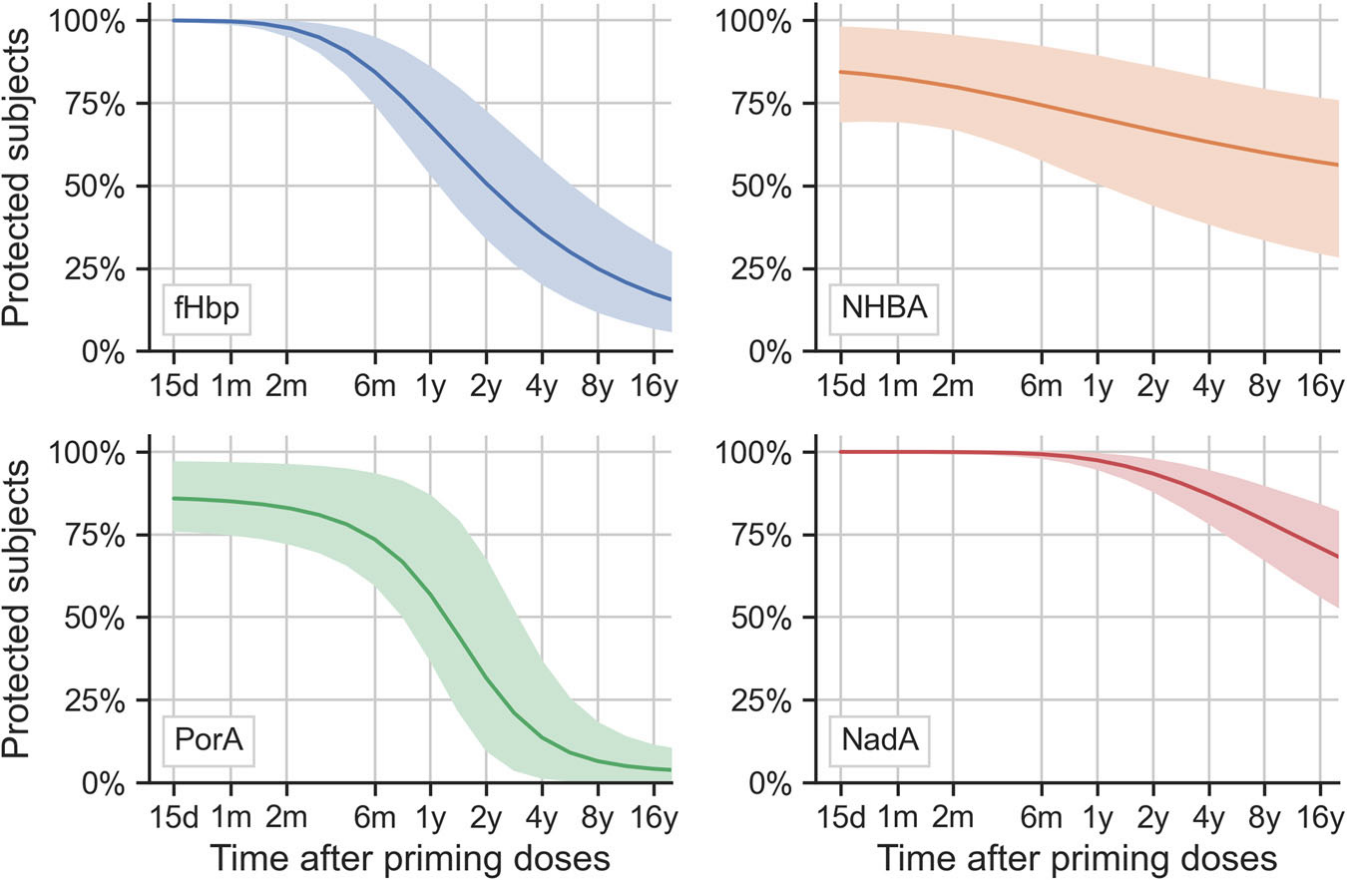
Predicted overall protection after priming vaccination, for each antigen-indicator strain. Best model’s predicted proportions of participants with hSBA titer ≥ 4 for each antigen, at different times after two priming doses of 4CMenB vaccine, in absence of booster vaccination. Posterior means are shown as lines, 95% credible intervals are reported as shaded areas and include both the inferred between-subjects and between-countries variability. d, day; m, month; y, year; fHbp, factor H binding protein; NHBA, Neisserial Heparin-Binding Antigen; PorA, Porin A; NadA, *Neisseria* adhesin A; hSBA, human serum bactericidal antibody; 4CMenB, four-component meningococcal serogroup B vaccine.

Overall, persistence differed by antigen, both qualitatively (curve shapes in Fig. 2) and quantitatively (Table 2). Protection elicited by PorA was the earliest and fastest to decline: at six months after the second dose the predicted proportion of protected participants was 73.5% [95% credible intervals (CIs): 60.0%; 93.3%], decreasing to 56.8% [37.1%; 86.6%] at one year and 31.6% [9.9%; 66.9%] at two years after vaccination. Persistence of protection by fHbp was higher: 84.4% [74.9%; 94.7%] after six months, to 68.2% [53.6%; 85.6%] at one year and 50.7% [34.3%; 72.2%] at two years after vaccination. NHBA protection was lower than fHbp at six months (74.4% [58.1%; 91.9%]), but higher at one year due to a slower decline, persisting at 63.2% [38.7%;82.2%] after four years. NadA provided the highest protection, due to the very high initial post-vaccination titers, with protection of nearly 100% at six months (99.3%, [98.3%; 99.9%]) and 79.3% [67.5%; 89.4%] at eight years post-second vaccine dose.

**Table 2:** Overall proportion of participants protected over time after two priming doses and after third dose (booster), for each antigen

Time after last dose	Post-priming protection (%) [95% CI (%)]	Post-booster protection (%) [95% CI (%)]
**fHbp**		
6 months	84.4 [74.9; 94.7]	69.9 [30.2; 91.4]
1 year	68.2 [53.6; 85.6]	54.5 [17.3; 80.2]
2 years	50.7 [34.3; 72.2]	39.9 [9.8; 65.6]
4 years	35.9 [20.5; 57.2]	28.2 [5.6; 50.7]
8 years	24.9 [12.0; 43.5]	19.7 [3.3; 38.1]
**NHBA**		
6 months	74.4 [58.1; 91.9]	91.2 [72.5; 99.1]
1 year	70.6 [51.0; 89.1]	87.5 [63.9; 98.4]
2 years	66.7 [44.4; 85.8]	83.4 [55.7; 97.7]
4 years	63.2 [38.7; 82.2]	79.4 [48.7; 96.7]
8 years	59.9 [33.9; 79.0]	75.4 [42.5; 95.7]
**PorA**		
6 months	73.5 [60.0; 93.3]	78.3 [52.5; 92.3]
1 year	56.8 [37.1; 86.6]	62.1 [27.0; 83.8]
2 years	31.6 [9.9; 66.9]	35.2 [6.8; 60.6]
4 years	13.6 [1.6; 36.3]	14.4 [1.3; 31.2]
8 years	6.5 [0.5; 18.0]	6.6 [0.4; 15.9]
**NadA**		
6 months	99.3 [98.3; 99.9]	100.0 [99.8; 100.0]
1 year	97.5 [95.0; 99.4]	99.8 [99.3; 100.0]
2 years	93.4 [88.3; 97.6]	99.0 [97.6; 99.9]
4 years	87.1 [78.6; 94.2]	97.0 [93.6; 99.4]
8 years	79.3 [67.5; 89.4]	93.5 [87.2; 98.2]

fHbp, factor H binding protein; NHBA, Neisserial Heparin-Binding Antigen; PorA, Porin A; NadA, *Neisseria* adhesin A; CI, credible interval.

Overall predictions had wider credible intervals than by country predictions, as they incorporated between-country variability. Figure S1 shows that differences between countries were lower in magnitude when compared with differences between antigens, and mainly limited to a horizontal shift i.e., to anticipated or delayed declines in average protection levels.

### Post-booster protection does not depend on time between priming and booster

Booster vaccination differed between countries: in Australia, Canada, and Chile the booster was a third 4CMenB dose, while in Poland and the USA it was a multi-component investigational vaccine that also included 4CMenB (therefore, the B component was the same in all countries). Among the different tested models, the one that best fitted the single one-month-post-booster data point was one where hSBA titers were not dependent on the time intercurred between priming and booster, compared with linearly or log-linearly time-dependent models. Similarly to post-priming, a hierarchical model able to reproduce the substantial between-subject variability provided the best fit.

**Fig. 3:**
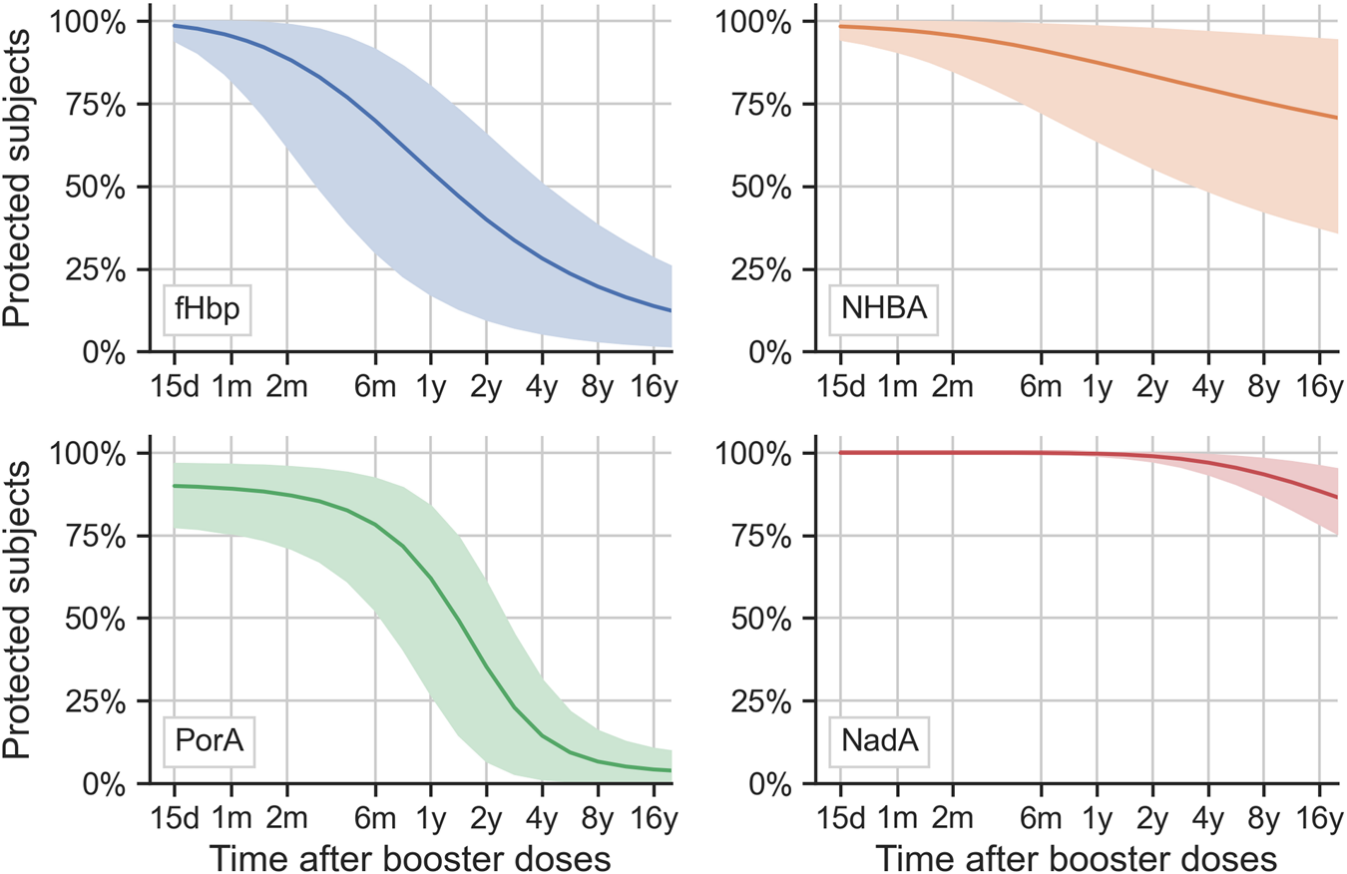
Predicted overall protection after booster vaccination, for each antigen-indicator strain. Best model’s predicted proportions of participants with hSBA titer ≥ 4 for each antigen, at different times after two booster doses of 4CMenB vaccine, extrapolated by applying post-priming fitted slopes to data taken one month after booster dose. Posterior means are shown as lines, 95% credible intervals are reported as shaded areas and include both the inferred between-subjects and between-countries variability. d, day; m, month; y, year; fHbp, factor H binding protein; NHBA, Neisserial Heparin-Binding Antigen; PorA, Porin A; NadA, *Neisseria* adhesin A; hSBA, human serum bactericidal antibody; 4CMenB, four-component meningococcal serogroup B vaccine.

Post-booster follow-up was only one month, and most data consisted of a single data point, thus, it was not possible to fit curves as done with post-priming data. Only post-booster intercepts were inferred. Then, post-priming slopes were re-used and applied to post-booster intercepts to extrapolate post-booster persistence of protection, while considering correlations between pre- and post-booster. Figure 3 and the second column of Table 2 show that persistence after a third dose (booster) varied particularly for NHBA and NadA when compared to post-priming, while smaller variations were observed for PorA and fHbp.

### Total persistence of 4CMenB protection for real-world MenB disease

**Fig. 4:**
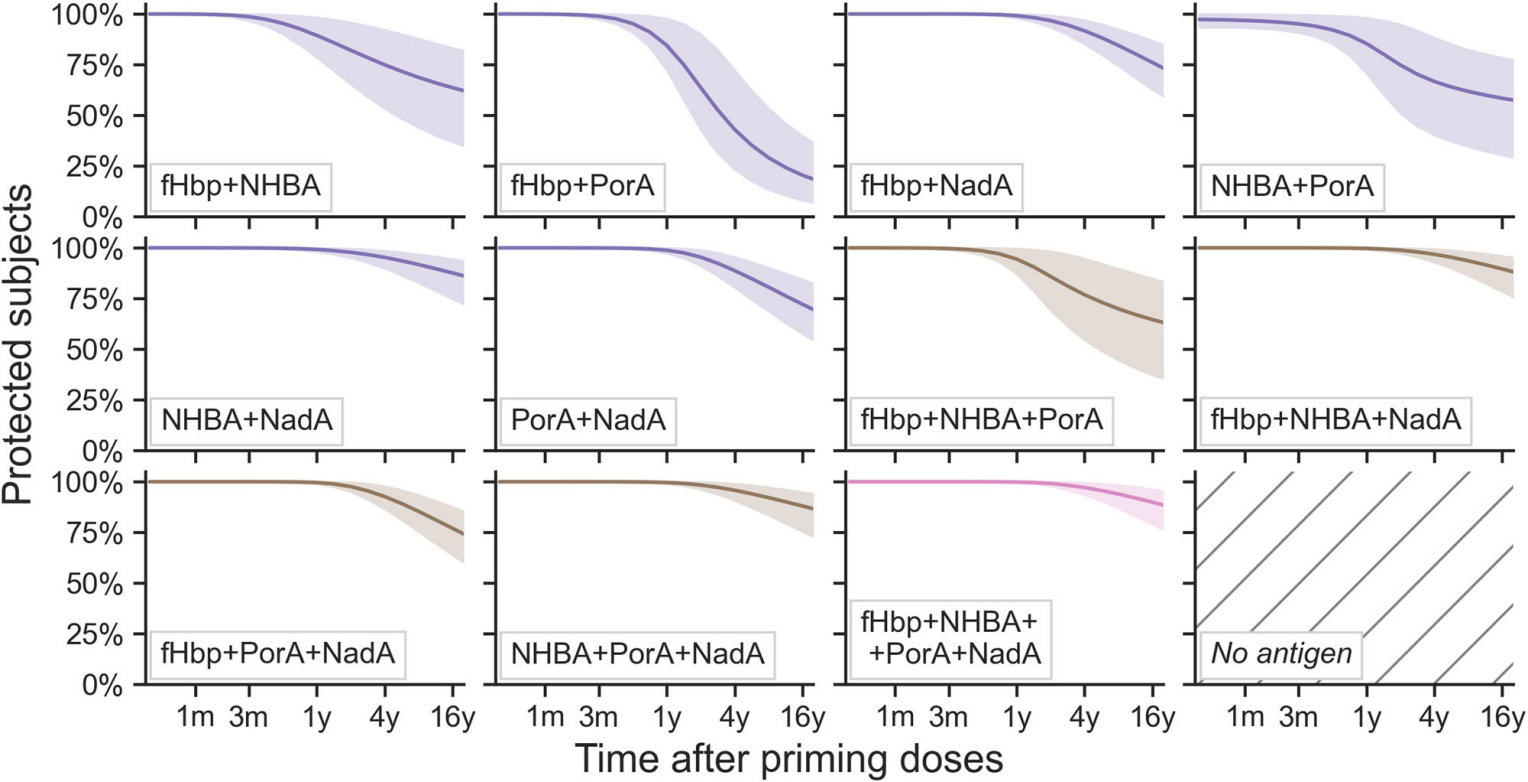
Persistence for MenB strain types predicted covered by more than one antigen after priming vaccination. Predicted proportions of participants with hSBA titer ≥ 4 for MenB strain types simultaneously expressing all eleven possible combinations of at least two antigens, with different colors (violet for two antigens, brown for three, pink for four), at different times after two priming doses of 4CMenB vaccine. At the bottom right corner, the last plot is intentionally left empty: MenB strains expressing none of the four 4CMenB antigens were considered not covered, hence no protection curve was derived. Posterior means are shown as lines, 95% credible intervals are reported as shaded areas and include both the inferred between-subjects and between-countries variability. m, month; y, year; fHbp, factor H binding protein; NHBA, Neisserial Heparin-Binding Antigen; PorA, Porin A; NadA, *Neisseria* adhesin A; hSBA, human serum bactericidal antibody; MenB, meningococcal serogroup B; 4CMenB, four-component meningococcal serogroup B vaccine.

Individual results for each antigen were combined assuming that bacterial killing elicited by one antigen is sufficient to provide protection, and considering that some participants may respond better to one antigen than to another (i.e., taking into account correlations between indicator strains). The persistence of protection for MenB strains harboring more than one antigen are shown in Fig. 4 for post-priming. There are 2^n^ = 16 combinations of *n* = 4 elements, thus, theoretically, 16 strain types in terms of the four 4CMenB antigens: four types expressing one antigen (Figs. 2 and 3), six with two antigens, four with three antigens, and one with all four 4CMenB antigenic components. The sixteenth type expresses no 4CMenB antigen, therefore, protection from such strain types was assumed null at any time.

**Fig. 5:**
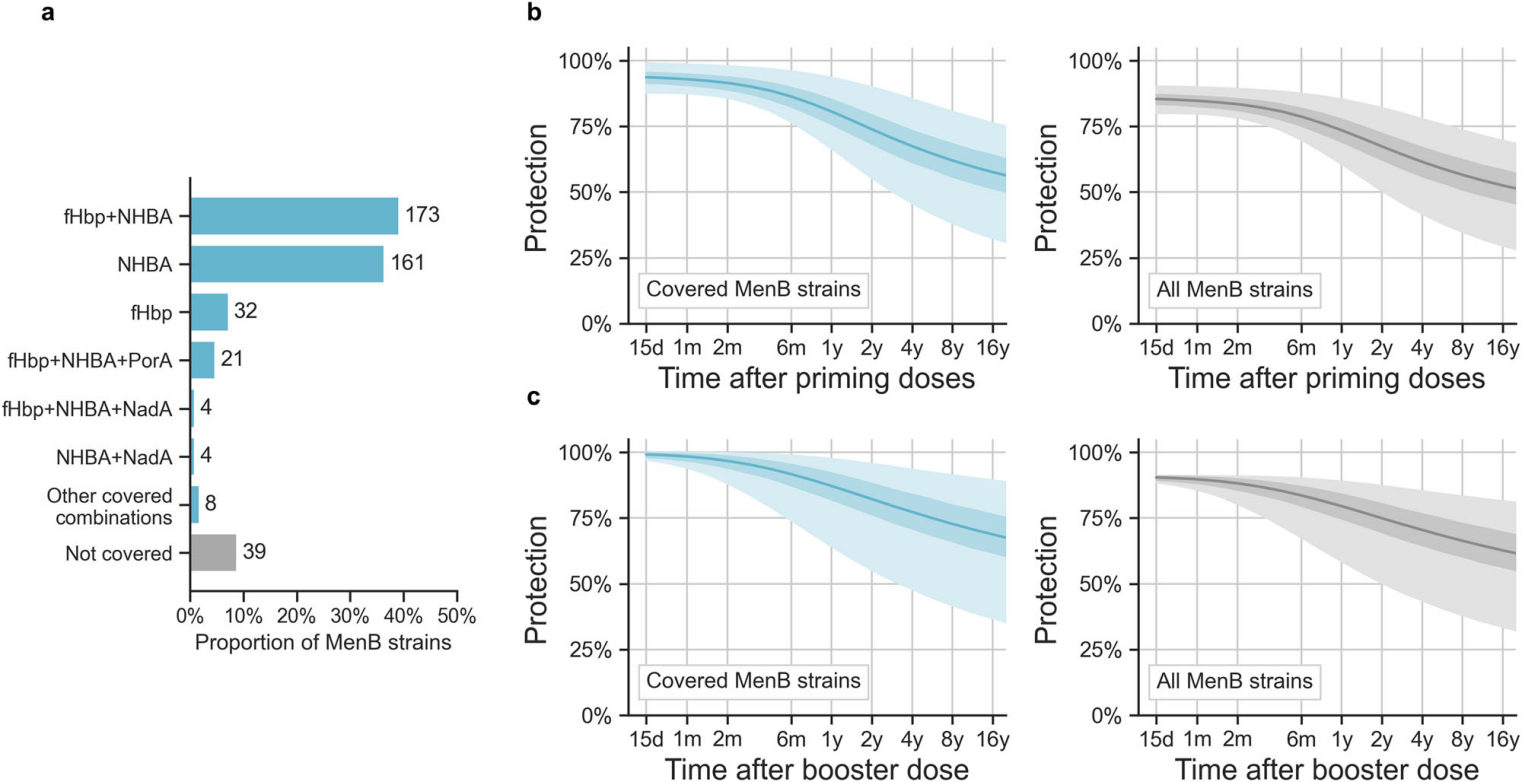
MenB strain typing and total 4CMenB persistence of protection. Panel **a** reports the prevalence of each MenB strain type in correspondence to 4CMenB antigens detected in a collection of 442 MenB strains representative of USA epidemiology. **b**, **c** report, respectively, post-priming and post-booster predictions, 4CMenB persistence of protection after integrating the antigen-based results over MenB strain types, weighted by their prevalence shown in panel **a**. The prevalence was calculated on covered strains only (*n* = 403 4CMenB-preventable strains, left side turquoise curves) and on all MenB strains (*n* = 442, including strains that are not covered by the vaccine, right side gray curves). The plots report posterior means as solid lines, 95% CIs for the posterior means as dark shades, 95% CIs for the sample predictions (that include variability by participants and countries) as lighter shades. d, day; m, month; y, year; fHbp, factor H binding protein; NHBA, Neisserial Heparin-Binding Antigen; PorA, Porin A; NadA, *Neisseria* adhesin A; n, number; USA, United States of America; CI, credible interval; MenB, meningococcal serogroup B; 4CMenB, four-component meningococcal serogroup B vaccine.

The best predictions for all 16 antigenic types were combined using 4CMenB typing data collected in the USA, where a panel of 442 pathogenically relevant MenB strains were characterized in terms of their potential to be killed (yes or no) by each of the four antigens (MATS coverage)^20^. Of the 16 theoretically possible antigenic types, only 11 were observed, and the four most prevalent types represented almost 88% (*n* = 387) of the strain panel^20^: fHbp-NHBA, NHBA alone, fHbp 1.1 alone and fHbp-NHBA-PorA (Fig. 5a). Less than 9% of the MenB strains were not covered by 4CMenB, being not sufficiently cross-reactive to any 4CMenB antigen as per the MATS.

Panel b and c in Fig. 5 report the predictions after integrating persistence of protection provided by each MenB type, weighted by their prevalence in the USA strain panel, considering all the MenB strains or only the 4CMenB-type strains (excluding the 39 non-covered strains), using post-priming and post-booster predictions. The mean proportions of protection from, respectively, 4CMenB-covered MenB strains and all MenB strains, were 80.7% [95% CI 66.6%; 93.7%] and 73.5% [60.7%; 85.4%] one year after priming, decreasing to 67.5% [45.7%; 85.5%] and 61.5% [41.7%; 78.0%] four years after priming. After the booster dose, protection was higher i.e., respectively, 87.2% [64.2%; 97.6%] and 79.5% [58.6%; 89.0%] after one year and 77.3% [47.9%; 93.6%] and 70.5% [43.7%; 85.3%] after four years (Table 3).

**Table 3:** Overall 4CMenB protection

Time after last dose	Post-priming protection (%) [95% CI (%)]	Post-booster protection (%) [95% CI (%)]
**4CMenB-types MenB strains**		
6 months	86.4 [76.7; 96.1]	91.7 [74.1; 98.9]
1 year	80.7 [66.6; 93.7]	87.2 [64.2; 97.6]
2 years	73.9 [55.3; 90.1]	82.2 [55.2; 95.8]
4 years	67.5 [45.7; 85.5]	77.3 [47.9; 93.6]
8 years	62.0 [38.2; 80.7]	72.8 [41.7; 91.4]
**All MenB strains**		
6 months	78.7 [69.9; 87.6]	83.6 [67.6; 90.2]
1 year	73.5 [60.7; 85.4]	79.5 [58.6; 89.0]
2 years	67.4 [50.5; 82.1]	74.9 [50.3; 87.3]
4 years	61.5 [41.7; 78.0]	70.5 [43.7; 85.3]
8 years	56.6 [34.9; 73.6]	66.4 [38.1; 83.3]

*CI* credible interval, *MenB* meningococcal serogroup B, *4CMenB* four-component meningococcal serogroup B vaccine.

## Discussion

In this study, for the first time antigen-specific data for the meningococcal correlate of protection were integrated with typing data to predict vaccine-induced protection from real-world MenB disease in the USA, showing that the persistence of protection provided by the multicomponent vaccine 4CMenB is more than persistence of each of its components alone. A flexible data-driven approach was used to find a model able to accurately reproduce longitudinal serological data collected from several clinical trials in adolescents and spanning up to eight years post-primary vaccination. The analysis provides detailed estimates of the persistence of protection for each of the four antigenic components included in the 4CMenB vaccine, after primary vaccination and after the booster dose.

Looking to single components, long-term persistence of protection following two primary doses of 4CMenB in adolescents was highest for NadA and NHBA antigens, followed by fHbp and PorA. NadA had the highest mean intercept in the model i.e., a stronger initial response. NHBA had the smallest power-law slope parameter, thus protection declined slower than for other antigens. fHbp persistence was lower but also followed a power law model, while the best performing PorA model was an exponential model.

Power law and exponential have previously been used to model longitudinal antibody data from different viral and bacterial diseases and respective vaccines^9,21,22^. In this work, we compared several variations of models that incorporate power laws and exponentials to find a best model. Moreover, through model selection, an important immunological property was implicitly tested: whether protection may be mediated by long-lived plasma cells that continuously replenish antibody repertoires and are key to long-lasting protection^8,9^. Indeed, antibodies decay nearly exponentially with a fixed decay rate, so that they persist for few days or weeks. Power laws, instead, emerge when assuming that antibody decay rates are not fixed, but heterogeneous and gamma-distributed, as previously shown^21^. Therefore, power laws indicate that there is additional and larger variability in antibody decay, that could be due to heterogeneity in persistence of antibody-generating plasma cells, that can last for months, years and even decades. NHBA, NadA and fHbp were all fitted by power law models.

For PorA, instead, the best-fitting evolution model was an exponential, indicating no or small additional heterogeneity in antibody lifetime. The lower persistence of PorA and the possible lack of long-lived plasma cells confirm that meningococcal vaccines based on OMVs have a relatively shorter duration of protection than conjugated vaccines^23^. However, the score achieved by a power law model was not significantly worse, thus PorA may generate some longer-lived plasma cells, but relatively fewer compared to the other antigens. Moreover, meningococcal OMVs are also known to have adjuvant properties, therefore, they may improve persistence of the other three components that are recombinant proteins^24^. A possible model that lies between the power law and exponential and, possibly, considers adjuvant properties of OMVs could be explored in future to verify these hypotheses.

The best-fitting model was hierarchical in both intercept and slope parameters: it accounted for between-subject variability in both immediate immune response and persistence of protection, that are not explainable as exclusively related to random fluctuations of the measured titers. This finding may suggest that response to vaccination widely varies between participants not only in terms of quality and quantity of antibodies, but also in terms of quality and quantity of long-lived plasma cells, that are responsible for the variation in power-law slopes.

The clinical data included hSBA titers measured up to one month after a booster dose. By comparing different post-booster models, we tested whether the immune response depends on time between the second of the two priming doses and the booster dose and found that it most likely does not. This may be an important indication that good long-lasting memory B cells have been generated at priming. Model-based predictions suggest that protection is slightly increased after a booster dose.

4CMenB vaccine’s correlate of protection—bactericidal activity—is measured on four antigen-indicator strains, each chosen because it is cross-reactive to only one antigen and not to the other three. The aim was to possibly have a unique estimate of the persistence of protection, but simply combining the four time-series of hSBA titers was not possible, as the antigens differed on many aspects, including their biological function as reflected by the differences in the best-fitted evolution models. Moreover, there are hundreds of variants of MenB strains. Some cannot be covered by any 4CMenB antigen, some express one antigen, like the indicator strains, while others co-express two or more 4CMenB antigens. This study represents a first attempt to provide a unique measure of protection elicited by 4CMenB on one hand, while, considering the significant variability of MenB strain population, on the other hand. To connect clinical hSBA data for the four indicator strains to real-world MenB strains, an additional data source was employed: MenB typing data (MATS) for 4CMenB antigenic components, from a large panel of strains representative of MenB epidemiology in the USA. Two kinds of persistence curves were then derived: one for protection from any MenB strain and one for protection from 4CMenB-type MenB strains (i.e., excluding non-covered MenB). Both are weighted averages of the four antigens, weighted according to the frequency by which the antigens are found in real-world settings.

Model-based predictions were used here to estimate vaccine protection at timepoints not directly measured in the original clinical studies whose data were used to fit such models. This is an important limitation, especially when considering predictions extrapolating over the time range of the observed data. However, we compared several models and chose the best performing as per WAIC metrics, whose predictions are a good fit to the clinical data, for all the antigens and in all the countries, as shown in Fig. 1. Another limitation was that one data point was available after booster dose (one month after booster dose), therefore, it was assumed that post-booster hSBA titers of the four antigens would follow the same evolution models inferred from post-priming data, with the same slopes (but different intercept). Furthermore, in Poland and the USA, the booster dose consisted of a different investigational vaccine that included 4CMenB antigens. Therefore, post-booster extrapolations are less reliable than post-priming predictions.

**Fig. 6:**
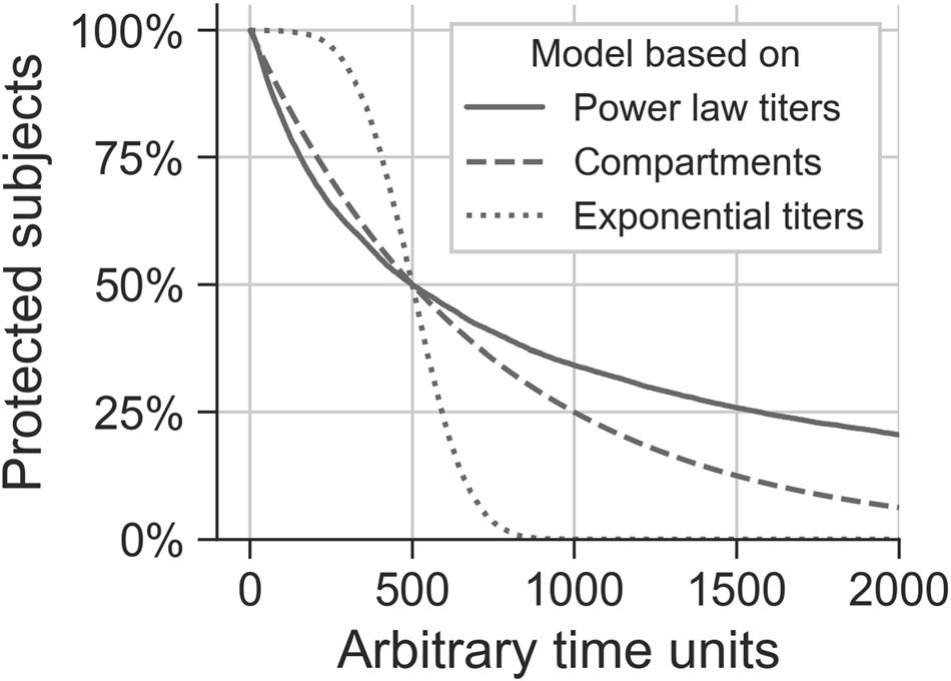
Comparison between outcomes based on different models. In this simple experiment, the predicted proportions of protected subjects based on two evolution models fitted to bactericidal titer data (exponential, in dotted line and power law, in solid line) are compared with the same outcome generated through a general compartmental model (dashed line). For all three models, the curves were produced by adjusting decay rates to predict 100% protected subjects immediately after vaccination, and 50% at time *t* = 500 (time arbitrarily chosen, so that units could be days, weeks, or any other time unit).

The persistence models developed here were fitted to clinical data from adolescents, but the same procedure may be followed in future for infants. Moreover, these models could also help to improve epidemiological compartmental models. Compartmental models of MenB disease and vaccination are used to evaluate vaccine impact and effectiveness for public health purposes, because they can account for both direct protection and herd immunity effects^25–28^. Compartments of vaccinated report the number or the proportion of immunized subjects at a given time. However, given the lack of evidence so far, waning immunity has been usually implemented in a simplistic way that leads to the exponential decline of the proportion of subjects protected by IMD. In this work we showed instead that, even if protection at individual levels declined exponentially as we found for PorA antigen, it appears unrealistic that the population level protection (a percentage of protected subjects) could decline exponentially. Figure 6 displays the outcomes of a simple computational experiment, highlighting the substantial differences in predictions generated by the three models. This scenario assumed that the only known information is that at a specific time (t = 500 time units), the proportion of protected subjects is halved with respect to an initial condition where the entire population was protected. If antibody titers declined exponentially, the proportion of protected subjects would not decline exponentially; instead, it would follow an s-shaped curve, leading to a shorter duration of protection compared with the compartmental model. In contrast, if titers followed a power law decline, the predicted proportion of protected subjects would show a longer tail than the one produced with a compartmental model. Therefore, in the simple example considered here, the compartmental model would respectively overestimate and underestimate the actual long-term effects of the vaccine. Using the evidence derived from real-world data, curves similar to those shown in Figs. 2 and 5 could easily be implemented to adjust predictions from compartmental models reproducing 4CMenB vaccination campaigns, ultimately improving 4CMenB cost-effectiveness evaluation.

Modeling studies are helpful to better understand data, test hypotheses and derive useful evidence, especially when data are complex and produced in different clinical studies. A mathematical model which fitted clinical data well was employed to investigate the long-term protection provided by the multicomponent meningococcal 4CMenB vaccine in adolescents. Model-based predictions were combined with typing data to evaluate protection from real-world MenB strains in the USA. With a multi-component vaccine, such as 4CMenB, there is an advantage over single-component vaccines, as longer persistence of protection with some components ensure a higher overall vaccine impact. These results may help support decision-making on adolescent vaccination programs in different countries and could be useful to improve the accuracy of cost-effectiveness models.

## Methods

### Data

The persistence data were time series of hSBA titers from adolescents vaccinated with two doses (priming vaccination) of 4CMenB vaccine given 1 to 6 months apart in six phase 2 and phase 3 clinical studies (Table 4). Only participants that satisfied the following criteria were included: they received both the priming doses, and had at least two datapoints for at least one antigen, with the last occurring at least two years after priming. The number of data points varied across countries, from a minimum of 136 in the USA (17 participants)^16,17^, up to 1,929 in Chile (131 participants vaccinated with three different 4CMenB schedules^13–15^). The longest follow-up was for participants recruited in Chile, where data were collected up to 7.9 years after complete vaccination; the shortest follow-up was just over two years after a 0–2-months schedule for participants from Poland and the USA^16,17^. Participants from Australia (38 participants^12,15^) and Canada (106 participants^12,15^) received two doses given one month apart and were followed for more than four years.

**Table 4:** Summary of the data to which all the models were fitted

Study reference	Country	4CMenB priming schedule (months)	Number of participants with >2 years follow-up	Number of available data points	Follow-up before booster (years)	Booster vaccine
^12,15^	Australia	0–1	38	359	4.1–4.4	4CMenB
^12,15^	Canada	0–1	106	897	4.1–4.4	4CMenB
^13–15^	Chile	0–1, 0–2, 0–6	131	1,929	6.2–7.9	4CMenB
^16,17^	Poland	0–2	21	160	2.1–2.2	MenABCWY
^16,17^	USA	0–2	17	136	2.0–2.3	MenABCWY

*4CMenB* four-component meningococcal serogroup B vaccine, *MenABCWY* investigational vaccine combining 4CMenB vaccine with a meningococcal MenACWY-CRM conjugate vaccine, *USA* United States of America.

Post-booster data (one month after a booster dose, given at any time to subjects that had already received the two priming doses) was available for 98% (*n* = 306) of the participants with post-priming persistence data. Participants from Poland and the USA did not receive the same vaccine given at priming (4CMenB), but an investigational vaccine that incorporates 4CMenB in combination with a multivalent meningococcal MenACWY-CRM conjugate vaccine^16,17^.

hSBA is the correlate of protection of meningococcal disease and is used to evaluate the efficacy of meningococcal vaccines^10^. Bactericidal titers were obtained for four MenB indicator strains, each exhibiting one, and only one, of the four vaccine antigens, thus indicating immune response to each vaccine component. Strain 44/76 was used as indicator for fHbp, strain 5/99 as indicator for NadA, strain M10713 (M07-0241084 for studies in USA and Poland) for NHBA, strain NZ98/254 for PorA. Titers were calculated as the reciprocal of a dilution (e.g., a dilution of 1:4 corresponds to titer 4). The lowest detectable titer was 2, values under the limit of detection were unknown and indicated as “<2”.

Only adolescents that received two doses of 4CMenB 1 to 6 months apart and with at least one hSBA result from a blood draw taken at least two years after the second dose were included in the analysis. Data from blood draws taken after booster doses were excluded. For each hSBA titer, time was calculated as number of days intercurred between second dose and blood draw.

All MenB typing data used here were derived from a distinct study where a panel of 442 strains (representative of MenB disease in the USA)^20^ were collected and tested using the MATS. This assay was developed to predict if a MenB strain is covered or not by 4CMenB, and by which specific antigens (coverage by one antigen being sufficient to predict bacterial killing)^20^.

### Mathematical models for the evolution of immune response over time

Several mathematical models were developed and tested to predict four outcomes i.e., the four time-series of hSBA titers against 4CMenB vaccine antigenic indicator strains, measured in sera from adolescents that received two vaccine doses. Candidate models were characterized by different levels of heterogeneity of immune response and different combinations of two models reproducing the natural evolution of antibody titers after vaccination.

The two antibody evolution models were the power law model and the exponential model, which have been widely used to reproduce the decline of antibodies elicited by vaccines against different viral and bacterial diseases, including human papillomavirus, hepatitis, pertussis, and meningococcal disease caused by different serogroups^21,22,29–31^. In the power law model, for each participant *i* the bactericidal titer *T* evolves over time *t* after the last dose as:

$${T}_{i}\left(t\right)={A}_{i}{\left(\frac{t}{{t}_{\min }}\right)}^{\!{b}_{i}},{\rm{with}}\,\,t\ge {t}_{\min }.$$

At time *t = t*_min_, *T* equals *A*. We set *t*_min_ = 15 days, so that *A* represents the bactericidal activity 15 days after vaccination, when its effect should be nearly at peak. Parameter *b* is the power law exponent that determines the evolution of *T* over time. Titer *T(t)* declines over time *t* for negative values of *b*. After log-transforming, the model becomes linear with respect to the logarithm of time *logt*:

1 $$log\, {T}_{i}\left(t\right)={a}_{i}+{b}_{i}\left({logt}-log\, {t}_{\min }\right),$$

where ${a}_{i}=\log {A}_{i}$ is the intercept of the corresponding *logt-*linear model.

In the exponential model, for each participant *i* the bactericidal titer *T* evolves over time *t* after the last dose as:

$${T}_{i}\left(t\right)={A}_{i}{e}^{{c}_{i}\frac{t-{t}_{\min }}{{t}_{\min }}}.$$

As for the power law model, *T* = *A* when *t* = 15 days after vaccination. Parameter *c* is the evolution rate of *T*, rescaled by *t*_min_. After log-transforming, the model becomes linear with respect to time:

2 $${\log T}_{i}\left(t\right)={a}_{i}+{c}_{i}\frac{t-{t}_{\min }}{{t}_{\min }}.$$

For this model, ${a}_{i}=\log {A}_{i}$ is the intercept of the corresponding *t-*linear model.

In the different models tested, some or all of the parameters *a*, *b* and *c* were fixed for all the participants, or varied between individuals in a hierarchical way (also called mixed effects model). In hierarchical models, the parameters $\theta =a,b,c$ were assumed to be normally distributed with mean ${\mu }_{\theta }$ and variance ${\sigma }_{\theta }$:

$$\theta \sim N\left({\mu }_{\theta };{\sigma }_{\theta }\right).$$

where $\theta =a,b,c$. Models are no more hierarchical if dispersion is removed by setting ${\sigma }_{\theta }$ to zero or, equivalently, by identically assigning $\theta =\,{\mu }_{\theta }$ to all the participants in a stratum. Moreover, model changes in performance were tested when implementing stratification by study country or not.

### Post-booster models

All the available post-booster hSBA titers were measured one month after booster doses. The post-booster model was developed as a continuation of the post-priming model, thus conditioned on the inferred post-priming posteriors, to account for correlations between titers after booster and post-priming response and persistence of protection. It required the introduction of one additional parameter.

Through model comparison, two different hypotheses were tested when analyzing data collected one month after booster. The first was that immune response depends on time intercurred between priming vaccination and booster dose. This was obtained by adding the additional parameter ${h}_{{\boldsymbol{i}}}$ to Eqs. (1) and (2), that is switched on after booster:

$${log\,T}_{i}\left(t=t^{\prime}\right)={log\,T}_{i}\left(t\right)+{h}_{{\boldsymbol{i}}}.$$

Where $t^{\prime}$ is one month after booster.

The second hypothesis is that immune response one month after booster is not dependent on the time that intercurred between priming and booster. This was modeled through a *k* parameter that is not summed to Eqs. (1) and (2), but substitutes them at one month after booster:

$${log\, T}_{i}\left(t=t^{\prime} \right)={k}_{i}$$

Moreover, three different levels of model hierarchy and stratifications for post-booster variance parameters *h* and *k* were evaluated, for a total of six models.

### Model fitting and evaluation

Titers were all logarithmically transformed before fitting. The models were Bayesian, with non-informative priors and left-censored normal likelihoods. The likelihoods were left-censored to allow evaluation even when titers were reported to be under the limit of detection, thus avoiding any a-priori imputation of titer data for undetected bactericidal activity that can introduce bias when fitting longitudinal data^32^. Bayesian full posterior distributions of model parameters were inferred with a Markov Chain Monte Carlo (MCMC) algorithm from the Python PyMC3 package^33^.

Models were compared through their expected log pointwise predictive densities calculated using the WAIC. A higher WAIC score indicates a model with better predictive accuracy^34^.

### Simulation of immunogenicity and persistence curves

Posterior distributions inferred from the best-fitting post-priming and post-booster models were used to generate immunogenicity curves after the two primary doses (in absence of booster) and after booster dose. For post-priming simulation, antigens log titers were simulated to evolve as in Eqs. (1) or (2), depending on model best fit antigen data. Post-booster log titers were simulated assuming to evolve in the same way as post-priming, but starting from a one-month-post-booster intercept. In practice, since the best model was the one in which booster immunogenicity did not depend on time between priming and booster, *logT* followed Eqs. (1) or (2) (depending on which was antigen’s best model after priming) with $a$ substituded by $k$.

For both post-priming in absence of booster and post-booster, the procedure consisted of computationally simulating 1000 times the vaccination of 5000 hypothetical participants from each of the five considered countries, and their bactericidal titers against each antigen at different times. Synthetic data were first generated by country, then merged into “overall” data.

The simulated hSBA data were then used to calculate the proportion of participants with bactericidal activity against the four antigens at each time point, by country, and overall. Participants with an hSBA titer equal or superior to four were considered protected^10^. Overall proportions represent the expected value of the bactericidal activity, when combining evidence from different countries.

### From antigens persistence to vaccine persistence using MenB typing data

Since there are four 4CMenB antigens which can be co-expressed, there are 16 theoretically possible types of MenB bacteria, relative to the vaccine-preventability of the disease they may cause. Four types correspond to strains that harbor one and only one antigen (like the indicator strains used for the hSBA). Six possible types include strains predicted to be killed by two antigens e.g., type fHbp-NHBA. Another four types are covered by three antigens (e.g., fHbp-NHBA-PorA), and one by all the four 4CMenB antigens. Finally, the sixteenth type simply includes all the MenB strains not covered by the vaccine, thus those MenB bacteria expressing no antigen corresponding to 4CMenB.

Bacterial killing (binary outcome) for the 15 covered types were derived at each time point for each simulated participant, in each country, for each posterior trajectory, assuming that a titer over threshold for one antigen is sufficient to elicit killing. For example, considering only two antigens for simplicity: if one year after vaccination a participant has an fHbp titer of 3 (<4, therefore not enough for killing) and an NHBA titer of 5 (enough for killing), the participant will be protected from NHBA and fHbp-NHBA types, but not from fHbp types, and of course not from “no-antigen” types. If after some time NHBA also falls below the protective threshold, the participant is no longer protected from any strain. This criterion was systematically applied to all simulations and derived immunogenicity curves (proportion of protected participants) for each of the 15 types. The sixteenth type has no curve, as there is no protection.

The prevalence of MenB types assessed in the USA panel of 442 strains was then used to weight such curves by the relative abundance of each strain type (with or without including the non-covered types, representing 8.8% of the panel)^20^. The sum of the weighted curves represents the final 4CMenB persistence, at each time point after vaccination. This 4CMenB-typing-based weighted average combines the previously calculated antigen persistence curves. The procedure was repeated, including and excluding non-covered types. In the first case, the results show protection against any MenB strain, while in the second case, they represent protection strictly from 4CMenB-preventable MenB strains. The same approach was applied for both post-priming and post-booster persistence.

### Comparison with a compartmental model

The predicted proportion of protected subjects over time based on an assumed antibody titer evolution model (either exponential or power law), was compared to a compartmental model. In scenarios where longitudinal data on antibody titers are not available, the persistence of protection is usually assumed, such as by estimating the time at which the proportion of immunized subjects is halved. A commonly used model to predict the proportion of immune subjects over time is a SIR-like (Susceptible–Infected–Recovered) compartmental model that includes vaccination. This model can be described by the following differential equation (for simplicity, only the vaccinated compartment *V* is reported):

$${dV}/{dt}=-{wV}$$

where the variation in the number of immunized subjects *V* over time is proportional to *V* itself, multiplied by a rate of waning immunity *w*. The solution of this equation is an exponential decay for the protected population. This is distinct from the exponential titer evolution model (Eq. 2), which does not generally correspond to an exponentially declining protected population.

To allow a direct comparison between models, all data were computationally generated for the same model population of 10,000 vaccinated individuals, with two constraints: i) following vaccination, 100% of the population is initially protected, and ii) after an arbitrary time of 500 time units (which could represent days, weeks, etc.), the proportion of the protected population declines to 50%. Specific details regarding the three models are provided below.

The population was composed of 10,000 immunized subjects, with 50% no longer protected after 500 time units.

For the two evolution models, 10,000 antibody titers were generated at time *t*_min_ = 1 using Python’s random generator for the lognormal distribution (parameters: mean = 5.03 and sigma = 1). The protective threshold was set to log(4). Given the randomly generated titers, at time *t*_min_ 9998 subjects were over threshold (99.98%). Titers for *t* > *t*_min_ were then generated using Eqs. 1 and 2, with the titers at *t*_min_ serving as the intercept and computationally solving for the slope parameter at which the proportion of titers over a protective threshold of log(4) was exactly 50% at time *t* = 500 units (Table S1). The solutions found for the rate and exponent parameters were, respectively for exponential and power law evolution models, *c* = 0.007311 and *b* = 0.5882.

For the compartmental model, two compartments were used to represent the number of unprotected subjects *U* and the number of protected subjects *V*, with *U* + *V* = 10,000 at any given time. The initial number of protected subjects was set to *V*(*t*_min_) = 9,998, to match the two evolution models. The waning parameter *w* was determined such that the proportion of protected individuals at time 500 would decrease to 50% (i.e., *V*(*t* = 500) = 5000). The resulting waning parameter was *w* = 0.001388.

## Supplementary information

Supplementary information

## Data Availability

All the data used in the models were from published studies.
