# Supplementary material for: Modeling the persistence of 4CMenB vaccine protection against real world meningococcal B disease in adolescents
Source: NPJ Vaccines. 2024 Dec 2;9:239. doi: 10.1038/s41541-024-01025-5 (PMC11612355; doi:10.1038/s41541-024-01025-5)
Supplement: Supplementary file 1 — Supplementary information [file 41541_2024_1025_MOESM1_ESM.pdf]

SUPPLEMENTARY INFORMATION

Manuscript title: Modeling the persistence of 4CMenB vaccine protection against real world meningococcal B disease in adolescents

Journal: npj Vaccines

Authors: Lorenzo Argante[1*], Ottavia Prunas[1,2], Duccio Medini[1,4], Ellen Ypma[3]

Affiliations:

[1] GSK, Siena, Italy.

[2] Swiss Tropical and Public Health Institute, Basel, Switzerland.

[3] GSK, Amsterdam, The Netherlands.

[4] Present address: Toscana Life Sciences Foundation, Siena, Italy.

***Corresponding author:** Lorenzo Argante

Address: via Fiorentina 1, Siena, Italy

Email: lorenzo.x.argante@gsk.com

Table S1. Summary of best model's inferred parameters

	Post-priming intercept a	Post-priming slope b	Post-booster intercept k
**Population means μ (mean and 95% CI)**			
fHbp			
Australia	5.1 [4.8; 5.5]	-0.95 [-1.08; -0.82]	5.1 [4.7; 5.5]
Canada	5.3 [5.1; 5.5]	-1.09 [-1.18; -1.00]	5.1 [4.9; 5.3]
Chile	5.9 [5.7; 6.1]	-0.90 [-0.97; -0.83]	5.6 [5.4; 5.8]
Poland	5.3 [4.7; 5.8]	-1.01 [-1.21; -0.80]	4.8 [4.3; 5.3]
USA	5.2 [4.5; 5.8]	-1.12 [-1.37; -0.88]	3.2 [2.7; 3.8]
NHBA			
Australia	2.1 [1.6; 2.6]	0.10 [-0.03; 0.23]	4.2 [3.8; 4.5]
Canada	2.5 [2.2; 2.8]	-0.01 [-0.09; 0.07]	4.2 [4.0; 4.4]
Chile	4.4 [4.1; 4.6]	-0.26 [-0.33; -0.19]	4.7 [4.5; 4.9]
Poland	3.4 [2.7; 4.1]	-0.44 [-0.65; -0.23]	4.3 [3.8; 4.8]
USA	2.8 [2.0; 3.5]	-0.42 [-0.66; -0.19]	3.2 [2.6; 3.7]
PorA			
Australia	2.6 [2.2; 3.1]	-0.0036 [-0.0047; -0.0026]	3.5 [3.1; 4.0]
Canada	2.5 [2.2; 2.8]	-0.0035 [-0.0043; -0.0028]	3.3 [3.1; 3.6]
Chile	4.2 [4.0; 4.4]	-0.0025 [-0.0030; -0.0021]	3.7 [3.5; 4.0]
Poland	3.0 [2.4; 3.6]	-0.0037 [-0.0049; -0.0026]	3.0 [2.5; 3.6]
USA	3.0 [2.3; 3.7]	-0.0059 [-0.0080; -0.0042]	2.4 [1.7; 3.0]
NadA			
Australia	5.6 [5.3; 5.9]	-0.52 [-0.64; -0.39]	7.3 [6.9; 7.6]
Canada	5.6 [5.4; 5.8]	-0.52 [-0.60; -0.44]	8.0 [7.8; 8.2]
Chile	6.7 [6.5; 6.9]	-0.69 [-0.75; -0.62]	7.6 [7.4; 7.8]
Poland	6.2 [5.7; 6.8]	-0.76 [-0.97; -0.56]	7.8 [7.3; 8.2]
USA	6.1 [5.5; 6.7]	-0.45 [-0.68; -0.23]	6.7 [6.2; 7.3]
**Population standard deviations σ (mean and 95% CI)**			
fHbp	0.79 [0.68; 0.91]	0.28 [0.26; 0.31]	0.87 [0.74; 1.01]
NHBA	1.13 [0.99; 1.28]	0.28 [0.26; 0.31]	0.78 [0.64; 0.91]
PorA	1.17 [1.05; 1.30]	0.0017 [0.0013; 0.0021]	1.02 [0.88; 1.17]
NadA	0.63 [0.51; 0.74]	0.28 [0.26; 0.31]	0.71 [0.57; 0.86]

Posterior means and 95% credible intervals are reported for each parameter of the fitting model: μ_a, μ_b, μ_k, σ_a, σ_b and σ_k. The best performing model was stratified by participants' country only for population means μ. Population standard deviations for slopes σ_b depend on evolution model (exponential for PorA, power law for the other antigens). In addition to these parameters, an error parameter was estimated: $\varepsilon = 0.80$ [0.77; 0.83].

fHbp, factor H binding protein; NHBA, Neisserial Heparin-Binding Antigen; PorA, Porin A; NadA, *Neisseria* adhesin A; CI, credible interval; USA, United States of America.

Figure S1. Predicted protection after priming vaccination, for each antigen-indicator strain and country

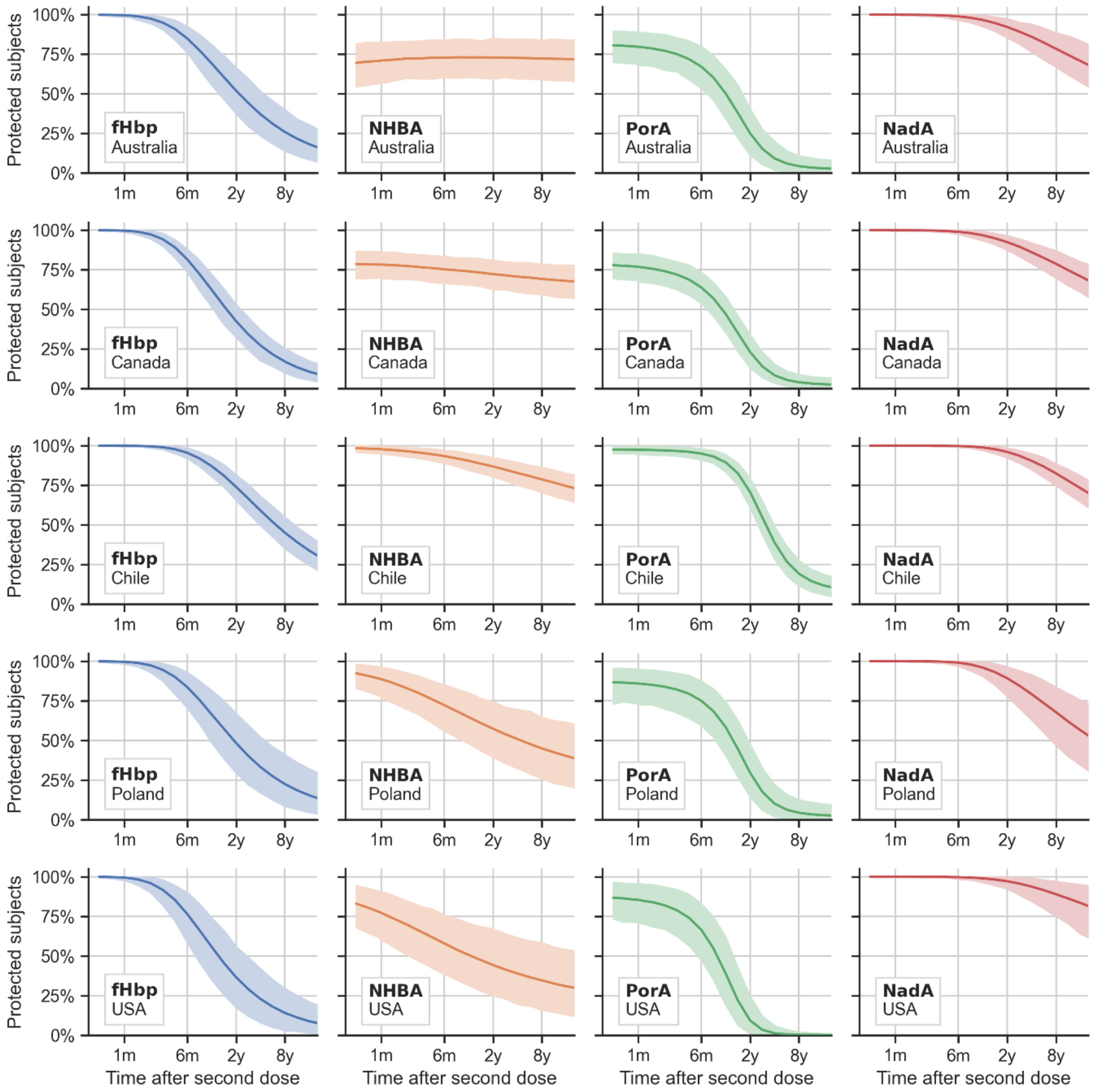

Country-based predicted proportions of participants with hSBA titer ≥ 4 for each antigen, at different times after two priming doses of 4CMenB vaccine, in absence of booster vaccination. Posterior means are shown as lines, 95% credible intervals are reported as shaded areas.

m, month; y, year; fHbp, factor H binding protein; NHBA, Neisserial Heparin-Binding Antigen; PorA, Porin A; NadA, *Neisseria* adhesin A; USA, United States of America; hSBA, human serum bactericidal antibody; 4CMenB, four-component meningococcal serogroup B vaccine.
